# Motivation of sex workers who provide camming services to engage in sex with their real-life and virtual partners

**DOI:** 10.3389/fpsyg.2023.1173902

**Published:** 2023-07-04

**Authors:** Anna Kelberga (Kelberg), Baiba Martinsone

**Affiliations:** Department of Psychology, University of Latvia, Riga, Latvia

**Keywords:** sexual motivation, reasons for sex, virtual sex, sex work, camming, chat hosts

## Abstract

In this study, the motivations of 80 sex workers who provide camming services (76 females and 4 non-binary/trans/gender fluid individuals; aged from 20 to 49 years, M = 30.68, SD = 6.43; 56.2% married or in a committed relationship, 18.8% in a non-committed relationship and 25% - single) were compared in terms of engaging in sexual activity with their real-life partners versus their virtual partners (predominantly kink-oriented clients). Presented with 16 reasons to engage in sexual activity, the respondents rated the frequency to engage in sex for each of these reasons with their real-life and virtual partners. Results showed that there were five reasons in which there were differences in motivation to engage in sex with real-life versus virtual partners and 11 reasons showed no differences. Specifically, respondents reported engaging in sex more often with their *virtual partners* to get resources and to experience a specific type of sex (kink), while they reported engaging in sex more often with their *real-life* partners to experience physical pleasure, motivated by physical desirability of a partner and to express love and commitment. However, for all other reasons that motivate people to engage in sex, including stress reduction, experience seeking, self-esteem boost, social status, revenge, utilitarian reasons, emotional expression, duty or pressure, thrill of the forbidden, mate guarding, and desire to have sex with a person of other gender, respondents engaged in sex equally frequently with their real-life and virtual partners (clients) and there were no statistical differences. This study adds to the existing research on sex work by providing insights into the motivations of sex workers to engage in sex with different partners and demonstrates that apart from common sense differences the reasons to engage in sex with clients and real-life partners are vastly similar.

## Introduction

Previous research has investigated motivation to engage in sex among various populations – college students (Meston and Buss, 2007), elderly adults (Wyverkens et al., 2018), women in casual and committed relationships (Armstrong and Reissing, 2015), lesbian, bisexual, queer, and questioning women (Wood et al., 2014), monogamous and non-monogamous individuals (Kelberg and Martinsone, 2021). Research by Kelberg and Martinsone (2022) has hypothesized that motivation to engage in sex is not a static and fixed characteristic of an individual but is contextual and motivation to engage in sex might vary with different partners. Drawing on the strategic pluralistic mating strategy theory, which posits that people engage in sex to fulfill different relationship needs (Gangestad and Simpson, 2000; Mogilski et al., 2017), the authors of this study investigate whether the motivation to engage in sex differs depending on whether a sex worker providing camming services engages in sex with their real-life partner or their virtual partner (client).

### Sex work

Sex workers are individuals who engage in sexual services in exchange for money or other goods. Harcourt and Donovan (2005) identified at least 25 types of sex work, that include, but are not limited to escort, street sex work, lap dancing, massage with sexual services, traveling entertainment, telephone/virtual sex, etc. These activities can be performed for a fee and be a primary source of income for an individual, or be an additional or occasional source of income (say, gifts) (Harcourt and Donovan, 2005). Estimating the prevalence of individuals involved in sex work is a challenging task as it varies across different countries. However, researchers have approximated that anywhere between 0.4 and 7.4% of the female population engages in transactional sex (Vandepitte et al., 2006). Some forms of sex work involve genital interaction (e.g., escort) and some do not (e.g., virtual sex or lap dancing) (Harcourt and Donovan, 2005). Sex work can take various forms, including those that involve direct money exchange (e.g., sex services at a brothel) and other forms of remuneration, like a paid vacation (e.g., traveling entertainers) or gifts that later may be exchanged for money (e.g., femmes libres) (Harcourt and Donovan, 2005).

This research focuses on the examination of “sex work,” which pertains specifically to consensual adult sexual labor. The study does not delve into the topic of illegal and non-consensual sex work, such as sex trafficking and forced sex. To emphasize the aspect of consent, some authors use the term “consensual sex work” to differentiate it from forced sex work. However, other authors argue against the usage of the term “consensual sex work” as they believe that providing sexual services without consent should not be considered as work, but rather as a form of assault or abuse (Language Matters: Talking about sex work Bruckert et al., 2013). In light of this, the authors of this study adopt the terminology used by McMillan et al. (2018) and Vuolajärvi (2022) and utilize the term “sex work” when referring to economically motivated sexual labor. McMillan et al. (2018) assert that this term is the most descriptive and practical, as it characterizes intimate exchange as a matter of work rather than a moral or ethical debate.

Camming is a digital live performance of sexual nature and includes a mixture of audio, video, and text interaction. This form of sex work amounts to 20% of the total pornography industry and is the fastest growing sector of sex industry (Patella-Rey, 2021). Sex workers who provide camming services, also known as chat hosts, models, or performers, offer their services consensually and voluntarily, and may be either individuals or couples who livestream their performances to viewers upon request (Falardeau, 2019). In contrast to conventional pornography, the act is not pre-recorded, but involves real-time interaction between the performer/s and a viewer/s (Henderson, 2011; Falardeau, 2019). Some chat hosts charge a rate per minute of live streaming, some earn for a performance and some earn money from selling their merchandize (Falardeau, 2019). Digital sex workers constitute a diverse and heterogeneous group. The majority of digital sex workers are women, although others are also involved in internet-based sex work, including men as well as transgender, non-binary, or gender-fluid individuals (Sanders et al., 2018). Many digital sex workers combine online work with other forms of sex labor (Sanders et al., 2018). According to Jones (2020), the camming industry is appealing to individuals who prioritize physical security and autonomy, with camming being a preferred option due to the perceived safety and reduced self-consciousness offered online, and its accessibility to those who may face barriers to other forms of sex work based on factors such as gender, race, sexuality, age, or disability, with online sex work offering benefits such as independence, flexibility, and control over the working environment. However, the field also has some drawbacks, such as the time required for marketing the services, also many find that it requires a significant amount of emotional involvement with a client (Sanders et al., 2020). Furthermore, online sex workers are not entirely immune to violence and harassment (Jones, 2015; Sanders et al., 2018).

### Reasons to engage in sex

In recent years, there has been a surge of research on human motivation to engage in sex. Early attempts to scientifically research reasons to engage in sex dates to the eighties and nineties when researchers topped obvious reasons of procreation, pleasure and tension release by a few more, like enhancing feeling of personal power and stress release (see Symons, 1979; Leigh, 1989; Hill & Preston, 1996). Subsequent studies have expanded the list of motivations to engage in sex and examined how sexual motivation varies depending on factors such as relationship type -long-term, short-term or extra-dyadic relationship (Buss and Schmitt, 1993; Gangestad and Simpson, 2000; Greiling and Buss, 2000). More recent research has both expanded the number of reasons to engage in sex (see Meston and Buss, 2007 for an exhaustive list of 142 reasons to engage in sex) and explored how sexual motivation is impacted by individual characteristics like gender (Kelberg and Martinsone, 2021), age (Meston and Buss, 2007; Wyverkens et al., 2018), relationship arrangement (Kelberg and Martinsone, 2021), partner status (Kelberg and Martinsone, 2022), commitment (Armstrong and Reissing, 2015), and sexual orientation (Wood et al., 2014). These findings highlight that motivation to engage in sex is not a fixed characteristic, but rather a complex and contextual phenomenon that is influenced by a range of personal factors (e.g., age, gender, etc.) and partner/relationship factors (e.g., partner status, relationship arrangement, commitment level to the partner, etc.), indicating that sexual motivation is not solely an individual attribute, but is also impacted by the specific characteristics of the partner and the relationship.

### Research aims

This study examines differences of sexual motives of sex workers who provide camming services to engage in sex with their real-life and virtual partners (clients). Extensive research in the past has thoroughly explored human motivations for participating in sexual activity, resulting in an expanded list of reasons considered, and taking into account demographic characteristics, relationship context, and individual relationship needs.

Meanwhile, most of the studies that investigate sex work, are focused on the prevalence of sexually transmitted infections (STIs) among sex workers and the measures taken to prevent their spread (for, e.g., see Steen and Dallabetta, 2003; Argento et al., 2019; Platteau et al., 2022); the issue of sex trafficking, examining its causes, effects, and potential solutions (for, e.g., see Gerassi et al., 2021; Cockbain et al., 2022; Motseki and Mofokeng, 2022); sexual abuse and exploitation in the sex industry, including the use of violence, coercion, and human rights violations (for, e.g., see UN Women, 2020; Navarrete Gil et al., 2021), also well-being of sex workers, including their physical and mental health (Romans et al., 2001; Beattie et al., 2020; Armstrong, 2021), as well as their economic and social status, and legal and policy issues governing sex work, examining their effectiveness in protecting the rights of sex workers and preventing exploitation (for, e.g., see Graham, 2017; Platt et al., 2018). However, little is known about sex workers’ motivation to engage in sex. The number of studies that investigated sex workers’ motivation to enter the sex industry is scarce. A study of UK students working as sex workers reports that while two thirds of study participants got involved in sex work to fund a particular lifestyle, a significant number of the study respondents engaged in sex work motivated by pleasure and flexible working hours (Sagar et al., 2015). Another study of German students that engaged in sex work, states that only a third of respondents work in sex industry primarily motivated by the possibility to obtain a higher income than in other jobs (Ernst et al., 2021). A study of adult Indian sex workers reported that their study participants were motivated to choose sex work versus other job opportunities as it provides them with more freedom and autonomy over their bodies, higher earnings, flexible hours of work, and flexibility to manage their dual responsibilities of a nurturer and provider (Sinha, 2015). While these studies confirm that monetary benefits are among the reasons to enter the sex industry, they highlight that the issue is complex, versatile, and nuanced. Motivations can vary, and factors such as lifestyle preferences, pleasure, flexibility, freedom, and autonomy are also significant considerations. It is important to acknowledge the diverse motivations of sex workers and recognize that the decision to engage in sex work is influenced by a range of individual and contextual factors. As one of the sex workers put it: “people tend to think that money is the sole reason *(why sex workers engage in sex)*” but authors of this study hypothesized that there is more.

In the present study, it was hypothesized that study participants would report higher levels of motivation to engage in sex with their virtual partners (clients) motivated by financial gain (resources). In the end, pay is one of the major characteristics that differentiates a job from other activities. However, if we put aside cases where sex workers are coerced into sex and focus on those who engage in sex work consensually and voluntary, concepts of organizational psychology can be applied to sex work. According to Herzberg’s (1968) Motivation Two-Factor Theory, the psychology of work motivation is complex and is influenced by many factors. Pay alone, as a “hygiene factor,” is usually not sufficient for an individual to be motivated to perform their work. Thus, it was hypothesized that chat hosts motivations for providing camming services go beyond merely financial incentives (resources). As the studied population works for a webcam platform that attracts kinky customers, it was reasonable to hypothesize that these sex workers would engage in sex more often with their clients than their real-life partners to experience specific kinds of sex, like kink. However, the authors are also interested in the intersection of motivations related to engaging in sex with real-life and virtual partners/clients, this way getting a deeper understanding of the complex motivations to engage in sex.

## Methods

### Participants

Participants of the study were 80 chat hosts that work for a Canadian adult website that offers one-on-one services such as adult chat, phone sex, sexting and video sex chat services. This platform positions itself as a kink-and fetish-friendly website. Age range among respondents was 20 to 49 years (M = 30.68; SD = 6.43). Majority of the chat hosts were in a relationship and 25% were single. A third of the respondents identified themselves as heterosexual (35%) and majority – bisexual (52.5%). 77.6% reported to have some sexual attraction both to males and females at least to some extent. 62.5% had previous sexual experience both with men and women. See Table 1 for detailed demographic information on study participants. Chat hosts were sent an invitation link to participate in the study by filling in a questionnaire. The email stated that the study is anonymous and voluntary.

**Table 1:** Demographic information of study paticipants, *n* = 80.

			*n*		%	
Gender	Other		4		5	
	Female		76		95	
Education	Less than high school degree		7		8.8	
	High School degree of equalivant		17		21.3	
	Some college but no degree		25		31.3	
	Associate degree		3		3.8	
	Bachelor’s degree		19		23.8	
	Graduate degree		9		11.3	
Relationship status	Married / Committed		45		56.2	
	Not Committed		15		18.8	
	Single		20		25.0	
Race -Ethnicity	Other		15		18.8	
	Asian / Pacific Islander		5		6.3	
	Black or African American		3		3.8	
	Hispanic		7		8.8	
	White / Caucasian		50		62.6	
Sex orientation	Other		8		10	
	Heterosexual		28		35	
	Gay or Lesbian		2		2.5	
	Bisexual		42		52.5	
Sexual attraction	Only to female		2		2.5	
	Mostly to females		4		5	
	Equally females and males		25		31.3	
	Mostly to males		33		41.3	
	Only to males		16		20	
Sexual behavior	Men only		30		37.5	
	Both men and women		50		62.5	
Age	**Min**	**Max**	**Mean**	**SD**	
	20	49	30.68	6.43	

### Measures

To explore the motivation of chat hosts to engage in sex with their virtual clients and real-life partners, authors used a modified YSEX? questionnaire (Meston and Buss, 2007), that was modified in the context of other studies for the purposes of non-monogamous populations (Kelberg and Martinsone, 2021, 2022) and demonstrated the questionnaire’s effectiveness in measuring the motivation of both monogamous and non-monogamous individuals. Chat hosts were asked to answer a set of 16 questions about their motivation to engage in sex with their real-life partners and the same set of questions in regards to their motivation to have sex with their virtual partners (a Likert scale from 1 to 5, where “1” was “none of my experiences” and “5” was “all of my experiences”). The question was presented to the respondents as follows: “People engage in sexual behaviors (i.e., sexting, masturbation, sexual intercourse) for many different reasons. Below is a list of some of these reasons. Thinking of your VIRTUAL/REAL-LIFE sexual experiences, please indicate how frequently each of the following reasons led you to have sex online/with your real-life partner/s” depending whether it asked chat hosts about their motivation to engage in sex with online or real-life partners. Procreation as a reason to engage in sex was removed from the questionnaire due to the virtual nature of work. In the context of previous studies, factor analysis indicated that each item is measuring a different dimension as each variable was loading heavily on a single factor only (Kelberg and Martinsone, 2022). See Table 2 for the full list of questions.

**Table 2:** Reasons to enagage in sex, 17 items of a modified YSEX? questionnaire.

1. Stress reduction: I wanted to release stress, anxiety, tension or to fight boredom
2. Pleasure: I was sexually aroused or wanted to experience physical pleasure
3. Physical desirability: The person was physically attractive
4. Experience seeking: I wanted new sexual experience or to act out a fantasy
5. Resources: I wanted to get resources from that person (such as promotion, money, etc.)
6. Procreation: I wanted to conceive a child
7. Social status: I wanted to enhance my social status or reputation
8. Revenge: I wanted my partner to feel jealous or hurt
9. Utilitarian reasons: I had sex for utilitarian reasons (such as burning calories, hoping to get rid of a headache or keeping warm)
10. Love and commitment: I wanted to feel connected to the person, express my love and commitment
11. Expression: I wanted to have sex in order to express my feelings such as being sorry, thankful, etc.
12. Self-esteem boost: I wanted to boost my self-esteem (such as feeling attractive or powerful)
13. Duty/pressure: I felt obligated or did not know how to say “no”
14. Mate guarding: I wanted to keep my partner from having sex with someone else.
15. Specific sex: “I wanted to have sex which I cannot have with my other partner (such as kink, fetish, anal, etc.)”
16. Another gender: “I wanted to have sex with a person of an opposite gender than my other partner.”
17. Thrill of the forbidden: “I wanted to experience the thrill of doing something forbidden.”

### Procedure

All chat hosts were sent an electronic invitation to participate in the survey *via* a group email, and their participation was voluntary and anonymous, with no personally identifying information or IP addresses tracked, and the data was collected using the SurveyMonkey survey tool. The survey included an informed consent form outlining the purpose of the study, confidentiality, voluntary participation, age restrictions, and researchers’ contact information, as well as demographic questions, core questions, and questions about the level of commitment to both real-life and virtual partners.

### Data analytic plan

Prior to the data analysis with SPSS 25.0 (IBM-SPSS Mac) software, the assumptions were checked. First, descriptive statistics (frequencies, means, and standard deviations) and ranking of reasons in the order of reported frequency were calculated. Next, to compare reasons of chat hosts to engage in sex with two groups of partners (virtual and real-life partners) the authors used paired samples t-tests to assess the significance of differences in the prevalence of various reasons for having sex. Effect sizes were evaluated using Cohen’s d metric. The power analysis has shown that the minimum detectable effect size for the power level of 80% and the 5% significance level reaches 0.32. A Bonferroni-adjusted significance level (alpha) of 0.003 (0.05 / 16 = 0.003125) was used to account for the multiple comparisons and control the overall family-wise error rate (Morgan, 2007). This significance level is also close to 0.005, recommended by Benjamin et al. (2018) to reduce the false positive rate in most fields. The results were robust to the choice of the statistical tests.

Ethical approval for the study was granted by the Ethics Committee for Humanities and Social Sciences research involving human participants, University of Latvia. The Ethics Committee of the University of Latvia operates under the framework of various legal acts and standards. It adheres to European Union and Latvian legal acts, including the General Data Protection Regulation (GDPR) and the Personal Data Processing Act. Furthermore, the committee adheres to ethics standards set by the European Commission, such as the European Charter for Researchers.

## Results

### Reasons of sex workers who engage in camming services to engage in sex with real-life and virtual partners

First, the study compared the primary reasons for sex among sex workers with their real-life partners and virtual partners. Top five reasons to engage in sex with real-life partners for sex workers are the following -pleasure, physical desirability of a partner, love and commitment, experience seeking, stress reduction. Top reasons to engage in sex with virtual partners (e.g., adult chat, phone sex, sexting and video sex chat services) for sex workers are similar to reasons to engage with their real-life partners – pleasure, physical desirability of a partner, experience seeking, love and commitment, followed by self-esteem boost (which is number six in ranking with real-life partners) (see Table 3 for a detailed overview of these findings). Obtaining resources from a partner comes as the sixth most frequent reason to engage in sex with their virtual partners. For a comparison, it is reason number 11 out of 16 reasons when it comes to sex with real-life partners.

**Table 3:** Differences in motivation to engage in sex with virtual and real-life partnets.

	Virtual partner			Real life partner			*n*	df	Paired samples		Cohen’s *d*
	Mean	Standard deviation	Rank order	Mean	Standard deviation	Rank order			t	*p*	
Pleasure	3.41	1.12	1	**3.83**	1.00	1	78	77	**−3.169**	**0.002**	**0.36**
Physical desirability	3.11	1.23	2	**3.58**	1.03	2	79	78	**−3.418**	**0.001**	**0.39**
Experience seeking	2.92	1.28	3	2.87	1.09	4	78	77	0.360	0.720	0.04
Love and commitment	2.89	1.19	4	**3.48**	1.15	3	80	79	**−3.654**	**0.000**	**0.41**
Self esteem boost	**2.88**	1.35	5	2.59	1.13	6	80	79	2.175	0.033	0.25
Resources	**2.76**	1.47	6	1.85	1.14	11	78	77	**5.641**	**0.000**	**0.64**
Stress reduction	2.68	1.02	7	2.80	1.14	5	80	79	−0.904	0.369	0.10
Thrill of the forbidden	**2.46**	1.20	8	2.20	1.14	8	79	78	2.266	0.026	0.25
Emotional expression	2.24	1.11	9	2.48	1.04	7	80	79	−1.679	0.097	0.19
Specific Sex	**2.01**	1.35	10	1.54	0.84	14	79	78	**3.543**	**0.001**	**0.40**
Duty or pressure	1.98	1.03	11	2.06	1.05	9	80	79	−0.961	0.339	0.11
Mate guarding	1.95	1.20	12	1.93	1.12	10	80	79	0.212	0.833	0.02
Other gender	1.94	1.09	13	1.81	0.99	12	80	79	1.296	0.199	0.15
Utilitarian reasons	1.68	0.93	14	1.78	0.93	13	79	78	−1.380	0.172	0.15
Social status	1.49	1.05	15	1.39	0.93	16	79	78	1.000	0.320	0.11
Revenge	1.39	0.77	16	1.43	0.86	15	79	78	−0.652	0.516	0.08

Significant differences are highlighted in bold. ^*^p < 0.05; ^**^p < 0.01; ^***^p < 0.001; Cohen’s criteria for small, medium, and large effects: small: 0,2, medium: 0,5; large: 0,8 or greater.

Then, the study compared the levels of motivation to engage in sex with real-life and virtual partners. To answer the question if there are differences in motivation to engage in sex with real-life and virtual partners, self-reported frequencies to engage in sex for various reasons with different partners were calculated and compared, Table 3 compares participants’ motivation to engage in sex with their virtual and real-life partner.

Chat hosts reported similar levels of motivation to engage in sex with their virtual and real-life partners across these factors: experience seeking, self-esteem boost, stress reduction, thrill of the forbidden, emotional expression, duty or pressure, mate guarding, other gender, utilitarian reasons, social status and revenge. For these factors, chat hosts did not show any significant difference in their level of motivation to engage in sexual activity with either their real-life or virtual partners.

However, significant differences were observed in other motivators to engage in sex. Participants reported engaging in sex with their real-life partners more frequently than with their virtual partners to experience physical pleasure (|d| = 0.36), as they found a person physically desirable (|d| = 0.39) and to express love and commitment (|d| = 0.41). In contrast, chat hosts reported more often to engage in sex with their virtual partners than with their real-life partners to obtain resources (|d| = −0.64) and to engage in specific types of sex, such as kink (|d| = −0,40).

## Discussion and implications

The aim of this study was to examine the underlying motivators that drive sex workers who offer camming services to engage in sexual activities with both their real-life partners and virtual partners (clients). The study contributed to a better understanding of the motivating factors that influence sexual behavior within this particular group, which has been relatively underexplored in this context.

The present study found that chat hosts were more likely to engage in sex with their virtual partners than with their real-life partners for two reasons: to obtain resources and to experience a specific type of sex. These findings were in line with the study’s hypotheses.

Pay (“resources” in the context of this study) is what differentiates a job from other activities, and it is reasonable that chat hosts engage in sex with their clients for financial reasons more frequently than with their real-life partners. However, it is noteworthy that pay ranked only sixth in the hierarchy of top motivators to engage in sex, following pleasure, physical desirability, experience seeking, love and commitment, and self-esteem boost.

Given that all of the chat hosts work for a webcam platform that caters to kink and fetish interests, it was expected that they would be more motivated to engage in specific types of sex, such as kink, with their virtual partners than with their real-life partners. However, there is possibly more -online platforms offer a certain level of anonymity and privacy, allowing individuals to engage in sexual activities without being easily identified or judged by others. This sense of detachment from real-life identities and the potential to explore specific sexual desires in a relatively discreet manner can contribute to increased motivation to explore one’s specific sexual interests with online partners versus real-life partners. Previous studies have indicated that the anonymity of the internet creates an environment conducive to sexual exploration (Ross, 2005; Albright, 2008) and online interactions offer individuals the opportunity to express their “true self” more freely compared to face-to-face relationships (Bargh et al., 2002). So, it might be that not only the kinky nature of the website that employs the chat hosts, but the anonymity of the internet itself that provides a sense of security and freedom for individuals to explore their specific sexual desires and fantasies without the fear of judgment or social repercussions, act as a motivation to engage in sex to experience a specific kind of sex.

Two reasons that stood out as more motivating to engage in sex with real-life partners than with their virtual partners (clients) was physical pleasure and physical desirability of a partner. Provided the nature of the virtual work, physical desirability for a partner and physical pleasure can be more easily achieved and experienced with real-life partners. Although sex work in cyberspace has more potential for pleasure compared to in-person sex work as it reduces the risks associated with traditional sex work (Jones, 2016), camming interactions with clients are generally more focused on meeting the client’s specific requirements and desires, rather than on personal pleasure (Warr and Pyett, 1999). In contrast, healthy consensual sexual interactions with real-life partners tend to be focused on mutual pleasure (Daker-White and Donovan, 2002).

Another reason that motivated chat hosts to engage in sex more frequently with their real-life partners than with their virtual partners (clients) was the expression of love and commitment. Studies have identified various factors that contribute to love and commitment in relationships. Tobore (2020) suggests that love is built upon factors such as attraction, connection, trust, and respect. On the other hand, commitment involves making a deliberate decision to choose one option over others and is rooted in the interdependence that develops between partners, the investment individuals put into the relationship, and the shared identity they develop as a couple (Stanley et al., 2010). These elements of love and commitment might be more characteristic to real-life relationships and contribute to the motivation for chat hosts to engage in sex with their real-life partners for this reason more often than with their virtual clients.

For all other reasons chat hosts engaged in sex equally often with their real-life and virtual partners (clients). While there are some differences in motivation to engage in sex with real-life and virtual partners that are discussed above, the majority of reasons, including the top most frequent reasons, are the same whether a chat host engages in sex with either their real-life partner or virtual partner (client). Saying that, it is important to note that the motivations to engage in sex are highly diverse, personal and multifaceted and can also be influenced by a sex worker’s personal circumstances, agency and control, the type of sex work, the setting, and etc. At the same time, findings of this study support a concept of *temporary bounded authenticity* introduced by Bernstein (2007) who suggests that modern day sex work in digital era is more authentic, oftentimes it engages eroticism, and emotional and physical connection, even if that exchange is temporarily.

### Limitations and future directions

This study gathered responses of 80 sex workers, a group of respondents that is usually hard to reach and has contributed to a better understanding of motivation to engage in sex among sex workers that provide camming services.

While in the past years there is more acceptance of sex work (Cao et al., 2015), there is a lot of stigma around it (Weitzer, 2018; Benoit et al., 2018a,b). Thus, it is difficult to say to which extent respondents of this study felt stigmatized and whether it has impacted their inclination to give more socially desirable responses.

The findings of this study should be applied to a broader group of sex workers with caution. Chat hosts engage in virtual sex, which not only does not involve direct physical contact, but also minimizes the risks of sex work associated with physical contact.

While it is clear that financial remuneration is a significant part of the transaction that happens between a sex worker that provides camming services and a client, it might be that there is more meaning to these relationships as they also give chat hosts similar benefits as real-life relationships. However, to get a broader understanding of sexual motivation, more research should be performed that considers nuances of sex work.

## Data availability statement

The raw data supporting the conclusions of this article will be made available by the authors, without undue reservation.

## Ethics statement

The studies involving human participants were reviewed and approved by the ethics committee of the University of Latvia. The patients/participants provided their written informed consent to participate in this study.

## Author contributions

AK conceptualized and designed the study, organized the database, performed the statistical analysis, and wrote the first draft and sections of the manuscript. All authors contributed to the article and approved the submitted version.

## Conflict of interest

The authors declare that the research was conducted in the absence of any commercial or financial relationships that could be construed as a potential conflict of interest.

## Publisher’s note

All claims expressed in this article are solely those of the authors and do not necessarily represent those of their affiliated organizations, or those of the publisher, the editors and the reviewers. Any product that may be evaluated in this article, or claim that may be made by its manufacturer, is not guaranteed or endorsed by the publisher.
